# Editorial comment to accompany *BJA Open* 100250

**DOI:** 10.1016/j.bjao.2024.100257

**Published:** 2024-01-24

**Authors:** Philip M. Hopkins

**Affiliations:** a Academic Unit of Anaesthesia, St James's University Hospital, Leeds, UK; b Leeds Institute of Medical Research, University of Leeds, Leeds, UK

It is the normal policy of *BJA Open* to require all systematic reviews (with or without accompanying meta-analysis) to have been prospectively registered with the PROSPERO database. As with the registration of clinical trials in a clinical trials registry, prospective registration of systematic reviews and meta-analyses is intended to improve transparency and minimise bias. Bias may arise, for example, if a protocol is changed after the start of a project: registries enable authors to record changes in the protocol along with a justification for doing so. This enables journal reviewers and editors, and ultimately readers, to evaluate changes in the protocol and form a judgment as to their impact on the rigour of the data presented and their interpretation.

The paper published in *BJA Open* by Booth and colleagues^1^ is a systematic review and meta-analysis of the airway management of adult epiglottitis that was not registered in PROSPERO. The history of this work is that it was originally formulated as a narrative review and submitted to our sister journal, the *British Journal of Anaesthesia*. This manuscript was not accepted for publication but there was a recommendation arising from the peer review process that there might be greater merit if the work was redone as a systematic review. This advice was acted upon by Booth and colleagues but, as they describe,^1^ they found that they were unable to register their systematic review and meta-analysis with PROSPERO because they had already initiated a large part of their search protocol in preparing the prior narrative review.

In accepting this work for publication, and considering the comments of peer reviewers, I have made a value judgment that it makes a useful contribution to the body of knowledge and the evidence base that may benefit clinicians dealing with the life-threatening emergency of adult epiglottitis. Throughout the process I have been struck by the openness of the authors and their efforts to minimise bias. By inclusion of a description of the evolution of this article in the Methods section of the paper and inclusion of the original protocol and subsequent amendments in the supplementary materials, I invite readers to form their own judgments as to the value of the data presented by Booth and colleagues^1^ and how it might impact their clinical practice.
